# Exploring antibiotic prescribing in public and private primary care settings in Singapore: a qualitative analysis informing theory and evidence-based planning for value-driven intervention design

**DOI:** 10.1186/s12875-021-01556-z

**Published:** 2021-10-15

**Authors:** Huiling Guo, Zoe Jane-Lara Hildon, Victor Weng Keong Loh, Meena Sundram, Muhamad Alif Bin Ibrahim, Wern Ee Tang, Angela Chow

**Affiliations:** 1 Department of Clinical Epidemiology, Office of Clinical Epidemiology, Analytics, and Knowledge, Tan Tock Seng Hospital, Singapore, Singapore; 2 Saw Swee Hock School of Public Health, National University of Singapore and National University Health Systems, Singapore, Singapore; 3 Division of Family Medicine, Yong Loo Lin School of Medicine, National University of Singapore, Singapore, Singapore; 4 National University Polyclinics, Singapore, Singapore; 5 School of Social and Health Sciences, James Cook University, Singapore Campus, Singapore, Singapore; 6 National Healthcare Group Polyclinics, Singapore, Singapore; 7 Lee Kong Chian School of Medicine, Nanyang Technological University, Singapore, Singapore

**Keywords:** Antimicrobial stewardship, Antibiotic prescribing, Primary health care doctors, Qualitative research, VALUE model for appropriate antibiotic prescribing in primary care

## Abstract

**Background:**

Singapore’s healthcare system presents an ideal context to learn from diverse public and private operational models and funding systems.

**Aim:**

To explore processes underpinning decision-making for antibiotic prescribing, by considering doctors’ experiences in different primary care settings.

**Methods:**

Thirty semi-structured interviews were conducted with 17 doctors working in publicly funded primary care clinics (polyclinics) and 13 general practitioners (GP) working in private practices (solo, small and large). Data were analysed using applied thematic analysis following realist principles, synthesised into a theoretical model, informing solutions to appropriate antibiotic prescribing.

**Results:**

Given Singapore’s lack of national guidelines for antibiotic prescribing in primary care, practices are currently non-standardised. Themes contributing to optimal prescribing related first and foremost to personal valuing of reduction in antimicrobial resistance (AMR) which was enabled further by organisational culture creating and sustaining such values, and if patients were convinced of these too. Building trusting patient-doctor relationships, supported by reasonable patient loads among other factors were consistently observed to allow shared decision-making enabling optimal prescribing. Transparency and applying data to inform practice was a minority theme, nevertheless underpinning all levels of optimal care delivery. These themes are synthesised into the VALUE model proposed for guiding interventions to improve antibiotic prescribing practices.

These should aim to reinforce intrapersonal ***V****alues* consistent with prioritising AMR reduction, and ***A****ligning* organisational culture to these by leveraging standardised guidelines and interpersonal intervention tools. Such interventions should account for the wider systemic constraints experienced in publicly funded high patient turnover institutions, or private clinics with transactional models of care. Thus, ultimately a focus on ***L****iaison between patient and doctor* is crucial. For instance, building in adequate consultation time and props as discussion aids, or quick turnover communication tools in time-constrained settings. Message consistency will ultimately improve trust, helping to enable shared decision-making. Lastly, ***U****se of monitoring data to track* and ***E****valuate* antibiotic prescribing using meaningful indicators, that account for the role of shared decision-making can also be leveraged for change*.*

**Conclusions:**

These VALUE dimensions are recommended as potentially transferable to diverse contexts, and the model as implementation tool to be tested empirically and updated accordingly.

**Supplementary Information:**

The online version contains supplementary material available at 10.1186/s12875-021-01556-z.

## Introduction

Antimicrobial resistance (AMR) is a rising global health threat. It has been projected that 10 million annual deaths would be attributable to AMR by 2050, with nearly half of these occurring in Asia [1]. Traditionally, antibiotic stewardship guidelines have primarily focused on tertiary hospitals, while such recommendations remain lacking in outpatient settings [2–4]. In 2016, the US Centers for Disease Control and Prevention (CDC) released a guiding framework for antibiotic stewardship in outpatient settings, which included primary care clinics, to extend monitoring and improvement of antibiotics use in such contexts [5].

Antibiotic prescribing itself has been described as an adaptive expertise, which requires the incorporation of clinical knowledge, experience and cognitive styles, but which is also framed by the characteristics of the patient [6]. Prescribing decisions have been found to be made under varying levels of support, cognitive loading as well as consideration of patient expectations, demands and self-presentation [6]. As such, the interplay between patient and doctor can be conceived as each adhering to practical considerations as well as social roles that influence their interaction. On the primary care doctor’s side, antibiotic prescribing has been shown to be dependent on their presentation of ‘expert self’, ‘benevolent self’ and ‘practical self’ during the clinical consultation [7].

Furthermore, the concept of value-based practice recognises the contribution of diverse values, from both patients and doctors, emphasizing the need to negotiate and align these to achieve shared decision-making in clinical practice [8, 9]. Patients’ values refer to what patients expect from their clinical experience while for doctors’, this includes the beliefs, both professional and personal, that determine priorities in clinical practice and related decision-making [10]. Organisational culture is also value-laden, driven by leadership and agreed standards of practice, that may override individual priorities and further influence doctors’ clinical decisions [11]. For example, it can be argued that doctors practising in an environment which shares, promotes and even monitors/evaluates against standardised best-practice guidance and information are more likely to learn to react in accordance to these.

Existing literature has emphasized procedural factors driving the doctor’s antibiotic prescribing, such as the lack of decision aids to support clinical judgment, diagnostic uncertainties and so forth [12–17]. Effects of clinical environment have been less considered [18–21], as have ways of consolidating the environmental aspects and procedural ones into a coherent synthesis or narrative. More evidence-based theory-driven approaches are needed to guide antibiotic intervention development that accounts for systemic differences that allows practitioners to judge how best to promote change in their own environments [22, 23]. Realist principles are applied to the current analysis [24]. Realist thinking seeks to account for diversity in *Context* while identifying *Mechanisms*, or aspects of practice which can be used to explain leverage positive *Outcomes* (CMO), in our case appropriate antibiotic prescribing.

Inappropriate prescribing is primarily defined herein, as that which is unnecessarily prescribed, meaning that no antibiotic was needed whatsoever unless otherwise specified. According to US CDC, this accounts for upward of 30% of prescribing rising to 50% when coupled with inappropriate selection, dosing and duration [25]. Social and behaviour change interventions to affect these outcomes have long been observed to occur at several social ecological levels which connect individuals to their institutional environments and wider communities or social norms [26]. In the current study, we have adapted this approach to frame our analysis starting with the intrapersonal, and drilling down to organisational, interpersonal levels, as housed in wider national and systems contexts.

This multi-level approach coupled with a focus on contrasting public and private primary care sectors, and filling the gap in understanding of systems where doctor both prescribes and dispenses medications in the clinic [12–21, 27–32] is seen to add to our knowledge gaps on complexity of antibiotic prescribing processes.

### Primary care structures in Singapore

In Singapore, 20% of primary care attendances occur in publicly funded polyclinics, with the remainder in private general practitioner (GP) clinics [33]. GP clinics include 1) Solo practices, which are single clinics that operate under a registered clinic name, 2) Small group practices, which refer to group practices that operate 2 to 8 clinics, and 3) Large group practices, which operate more than 8 clinics. On average, 20 to 30 doctors practise concurrently at each polyclinic on each clinic day, compared to one to two doctors at each private GP clinic. In most solo and some small group GP practices, diagnostic tests are outsourced to third-party service providers incurring additional operating costs whilst medications are dispensed by the doctor with no or minimal pharmacist involvement [34].

Doctors working in these practices are often the key decision-makers on how to operate their clinics. Large group GP practices, on the other hand, have a central operating structure that governs how clinics within the practice are run. This centralisation structure allows for operating cost-savings through successful negotiations of lower rental fees, and bulk purchases of diagnostic services and medications. In addition, GP clinics can also engage with varying third-party administrators (TPAs) or managed care organisations (MCOs) acting as middlemen to provide affordable care to employees of subscribing companies and sustainable patient referrals to participating GP clinics with different contract terms [35]. On the other hand, polyclinics are simply walk-in clinics accessible to all [34], but given their size and composition, they provide a wider range of multidisciplinary healthcare services than GP clinics. This includes outpatient services ranging from nursing care to pharmacy, radiology and laboratory services. Out-of-pocket payments by patients are much lower at polyclinics than GP clinics, due to government subsidies and economies of scale [36].

Given the complexity of how primary care practices are organised in Singapore, this study’s objectives are to explore the contexts, and related mechanisms behind decision-making processes for antibiotic prescribing by primary care doctors in Singapore by contrasting experiences across public and private sectors. In addition, we seek to summarise these findings into a conceptual model that accounts for the multiple socio-ecological levels that affect antibiotic prescribing across these settings using realist principles. In tandem, we relate the model to potential strategies for changes targeting primary care service improvement pertaining to appropriate antibiotic prescribing.

## Material and methods

### Study design and study population

Semi-structured interviews were conducted with primary care doctors in publicly funded polyclinics and private GP clinics between June 2018 and January 2020. In Singapore, there are 20 polyclinics and up to 2222 private GP clinics, serving a population of 5.7 million people [37]. To achieve maximum variation, the study participants were purposively recruited from four different settings: polyclinics, solo GP practices, small group GP practices and large group GP practices, with a good mix of age and years of practice in their current practice setting [38]. Locum doctors were excluded from the study. In addition to this maximum variation sampling strategy, the sample size required for this study was also grounded in the principles of data saturation [39]. The study was conducted and reported according to the Consolidated Criteria for Reporting Qualitative Research (COREQ) guidelines [40].

### Semi-structured interviews

A semi-structured topic guide was developed by HG (Female, MPH, Research Fellow) based on current literature and thereafter used to explore the factors influencing antibiotic prescribing practices among polyclinic and GP doctors in the primary care clinics. The topics and related questions explored the clinical and non-clinical factors that influenced participants’ prescribing practices at the intrapersonal, organisational, interpersonal and national or community levels (refer to the Topic Guide in Supplementary file (Additional file 1) for further details). Two sub-sections, one pertaining to specific decision-aids, the other a ranking exercise on community awareness interventions were not included in the current dataset. These have been removed from the appended topic guide.

Pilot interviews were conducted by HG with three primary care doctors to ensure content validity and the proper phrasing of questions in the guide. HG and another study team member (Female, MPH, Research Assistant), who were public health researchers trained in qualitative fieldwork, conducted the interviews. Interviews were also cross-audited by both study team members by observing three interviews respectively. The audit was undertaken to minimise interviewer bias, provide feedback on interview techniques as well as to ensure adequate probing and rapport building throughout the interview process.

Invitations letters were sent out to a number of primary care clinics and primary care doctors to invite them to the study. Interested clinicians were asked to contact the study team via email or text messages. The study team sought to ensure a good representation of age and years of practice before recruiting the participants. Recruited participants were provided with the participant information sheet and informed consent was taken onsite on the day of the interview. Each interview lasted for 45 to 60 minutes. Interviews were conducted at a preferred time and location specified by the participant to provide the greatest convenience and ease for the interview. Confidentiality was ensured by conducting the interviews behind closed doors at the respective doctors’ clinics or in a quiet corner in a public location. Interviews were conducted after consultation hours and in the absence of clinic staff from their respective clinics.

Moreover, no personal identifiers were collected, and participants were assigned a study identification number that was used throughout the study duration. Before the commencement of each interview, the interviewers would introduce themselves as researchers with no prior medical knowledge to ensure that participants could be candid and forthcoming with their responses. Every session was audio-recorded and transcribed verbatim.

### Data analysis

Data were analysed using an applied thematic approach [41], underpinned by realist principles [24, 42]. Two coders (HG and MABI) first read through all the transcripts to become familiar with the data and embarked on the process of organising it in Microsoft Word. Specifically, the coders compared the narratives driving the outcomes pertaining to in/appropriate antibiotic prescribing as shared by doctors across the publicly and privately funded primary care settings at different socio-ecological levels. This in-depth process informed the development of a preliminary codebook designed to help collate the data into broad areas for analysis according to our objectives. This task was based on a review of five transcripts from which saturation of the initial organising codes was achieved. The coders subsequently analysed the organised data independently, coding these manually (using comment boxes in Microsoft Word) for ongoing discussion.

Thematic codes were then derived iteratively as analyses continued. Discrepancies were discussed and resolved in the presence of a third study team member. Both intercoder agreement and saturation on emergent themes were systematically sought and achieved [41]. Saturation of themes was achieved faster on coding of polyclinic doctors’ data compared to GP data. This was judged to have occurred at about one-third way through polyclinic doctors interviews and more than halfway through for the GPs’ interviews. Agreed codes were applied to the full dataset by both coders before data reduction and summarisation were undertaken by one analyst (HG), who further refined the findings and supporting subthemes by grouping these and relevant illustrative quotes in Microsoft Excel. The relationships between the themes were then mapped out according to the levels that they targeted and summarised in a descriptive model (HG and ZH). Major themes are reported in **bold** as section headers while supporting subthemes are in *italics*.

Basic descriptive data of the participants was computed using STATA/SE 15.0 (StataCorp LLC, College Station, TX).

## Results

### Study participants

Thirty primary care doctors were interviewed. Their median age was 40 (range 27 – 69) years (Table 1). The majority were Singapore citizens and of Chinese ethnicity. There were more female participants represented in the polyclinics than GP clinics. In contrast, more participants from GP clinics had more than 10 years of clinical experience, and more had post-graduate training in Family Medicine than those from the polyclinics.

**Table 1:** Basic characteristics of participants

**Demographics**	**Polyclinics**	**GP Clinics**	**Total**
**Number of Participants**	17	13	30
**Age, in years**			
Median Age	35	47	40
Age Range	27 - 69	31 - 60	27 - 69
**Gender, N (%)**			
Female	13 (76)	5 (38)	18 (60)
**Ethnic Group, N (%)**			
Chinese	14 (82)	11 (85)	25 (83)
**Resident Status, N (%)**			
Singapore Citizen	15 (88)	13 (100)	28 (93)
Singapore Permanent Resident	2 (12)	0	2 (7)
**Highest Education Level, N (%)**			
Basic Medical Degree	10 (59)	5 (38)	15 (50)
Post-Graduate Degree in Family Medicine	7 (41)	8 (62)	15 (50)
**Total Duration in Medical Practice, in counts (%)**			
More than 10 years	7 (41)	9 (69)	16 (53)
**Total Duration in Current Practice, in counts (%)**			
More than 10 years	7 (41)	7 (54)	14 (47)

### Objective I: Exploring decision-making contexts for antibiotic prescribing across primary care settings

#### National level

##### Lack of standardised national antibiotic prescribing guidelines for primary care settings

In relation to the broader Singapore context, polyclinic doctors shared that they could refer to the paediatric dosing and disease-specific treatment guidelines made available to them by the polyclinics for antibiotic prescribing while these were generally absent in the GP settings, regardless of practice size. *While it was unanimous amongst polyclinic and GP doctors that guidelines had to be twinned with clinical judgment, the need for guidance was concurrently emphasized*. Since no national, standardised primary care ones existed, this meant that doctors often had to rely on multiple out-of-context sources (such as hospital and/or non-local guidelines) to inform their prescribing decisions:

*“… [while] Guidelines are only meant to guide us in a certain way but we still need our clinical discretion to decide whether truly the patient needs antibiotics or not.” (P5)*

*“In Singapore, I don’t think we have such a guideline. There’s a need for this* *[emphasis our own],* *but we don’t have [it]…Every single hospital, they have guidelines for antibiotics…we can follow [that].” (GP23)*

*“I rely more on Up-To-Date [referring to an international online clinical decision support resource] actually.” (P5)*

In sum, it was conveyed that a common set of nationally endorsed guidelines, tailored to primary care, was lacking and considered useful. Data suggested that consistency in use of these would provide a concrete starting point for aligning appropriate prescribing across primary care settings.

#### Intrapersonal level

##### Perceptions of types of care delivery and provider roles

Our data reflected *a tension between a desire to practise holistic healthcare provision* and an *expectation of customer service-oriented care delivery*:

*“For family practice to be able to continue and grow, acute care is not the main thing they have to focus on. Actually many times we actually look at the complete care…not just disease management…we are actually moving towards health prevention and disease prevention and…the next stage…health preservation, means how to actually make them healthier and better as a whole family, [and] not just the patient [alone].” (GP12)*

The latter occurred when patients presented more as clients than patients, and was more likely to result in giving into demands rather than supporting clinical judgment:

*“Healthcare has unfortunately become very customer service-oriented…after thoroughly counselling the patient…for indication, as opposed to having the patient scream at you…I may give Amoxicillin…it’s a fine line to tread between getting a complaint and exercising your best clinical judgment.” (P1)*

*“We still partially belong to the service sector you know, so a lot of times I do have to admit that if patients ask for antibiotics and they are insistent, our threshold to reject them is very low.” (GP22)*

Thus, we were alerted to the danger that primary care doctors, particularly in the private sector, may cave under insistent patient demands, rather than take the time to persuade them otherwise. Operational models and related factors shaped and perpetuated practice cultures that did not see the need to cave into patient demands; mechanisms that resulted in shared decision-making in particular helped to drive appropriate prescribing, related themes and subthemes are detailed below.

#### Organisational level

##### Role of operational models, practice size and shaping of organisational values

Primary care doctors are vulnerable to medical liabilities, yet our data revealed that *publicly funded operational model gave the doctors a sense of security due to perceived organisational backing*. This was said to contribute to relieving the pressure of having to satisfy patient demands:

*“When you are working in polyclinic[s], you have [the] government on your back…but in the private sector, the issue is when…medical legal [issues arise] …or patient come after you with a lawyer letter…when things happen, their backs are not covered.” (GP30)*

*“Practising here in the polyclinic gives me the liberty of not giving antibiotics unless its evidence-based and that, to a certain extent, gives me empowerment…not forced to give anything just to make the patient happy. So it’s easier to practise that way…you don’t have to bow to the wishes of the patient but you practise the way you are supposed to.” (P9)*

Furthermore, *in larger organisations -* like polyclinics or even large group GP clinics *- practice norms were established* *via* *shared protocols, and these were key to establishing shared values and continuity of preferred practices*:

*“A lot of times, it’s a legacy effect as well because for us, we belong to an organisation. So the clinic changes hands very frequently and usually when we come in, we inherit what was given to us. We make minor changes along the way but a lot of times we keep to what was given to us.” (GP22)*

*“We are more resource-strapped and we have more protocols, I feel that we are a bit more restricted when it comes to giving antibiotics.” (P20)*

Conversely, *standards of practice while tending to be less documented, shared institutional values were established by the leadership, and deviations were especially noticeable*:

*“In a place like us…you stick out…whatever thing you do will stick out…another doctor will pick up and…instant reporting…so that helps [to] keep us on our toe[s]…Whereas…if you are [in] solo [practice], it’s different. There’s nobody to police you…at least here…we will think…how would your peer[s] think…[and] what repercussion.” (P11)*

As such, size of the organisation and the legacy of values, or those of the current leadership, being placed either on shared protocols or undocumented yet ‘known’ practices formed the backdrop to antibiotic prescribing behaviours.

##### Effects of financing models on operations and related organisational values

The financing of clinics also helped to determine how clinics operated and these effects could trickle down to patient care. In polyclinics, the financing model is unified across all clinics and consultation fees are subsidised by the government for all local residents. *Polyclinic doctors shared that patient health financing schemes, insurance and claiming considerations had little or no influence on antibiotic prescribing habits since government-subsidy is provided to the majority of their patients*:

*“It doesn’t really matter for me because I feel that everyone is [given] subsidised care [by the government] … if I feel that antibiotic is really needed [rather than under prescribing], I will give the antibiotic. It doesn’t matter.” (P20)*

However, the situation in the GP clinics was much more complex due to potential third-party financing (TPAs) model for the patients. The opinions of solo and small group GPs towards TPAs or Managed Care Organisations (MCOs) acting as middlemen were mixed. Some GPs welcomed third-party financing structures because it sustained the patient pool. Others pointed out that *TPA and MCO arrangements came with contract restrictions that impacted clinical prescribing,* e.g. *it was described how with limitations on the* per capita *funding provided for each patient and the types of drugs claimable under the contract, doctors might resort to shortening the recommended antibiotic course and to prescribe only approved types of antibiotics to the patients*:

*“I don’t do company contracts [referring to TPA and MCO contracts] but I [have] work[ed] in company contract clinics before. So for example, you get antibiotics right, you give 5 days or 7 days. At the counter, the staff will cut down to 2 or 3 days because to cut costs…That’s why I don’t do contracts…they only give you this amount. So you either hit it or you bust it.” (GP14)*

*“We used to take up some of the third party insurance companies [referring to TPA and MCO contracts] and they will restrict you to prescribing generic rather than patented. But not the decision to prescribe or not to prescribe…For those who accept the insurance payments, the insurance company’s terms and conditions can be very restrictive…I think it affects one’s prescribing habits.” (GP25)*

Furthermore, GP doctors also felt that TPA and MCO contracts disrupted the patient-doctor relationship. This was especially problematic when the patient-doctor relationship was highly valued by GP doctors (to be illustrated below). In particular, *TPA and MCO contracts were perceived to turn the patient-doctor relationship into transactional cost-based one which lacked mutual trust or respect, and loyalty, creating a backdrop where antibiotics were more likely to be prescribed to satisfy patients’ demand*:

*“For those [under TPA/MCO] contract…there is no loyalty, there is no trust…there is no mutual trust or respect…So sometimes if you just want to get rid of the patient, you just give [antibiotics].” (GP14)*

*“Because the relationship you have with patients with managed healthcare [refers to TPA/MCO]… the patients already have a conception that you’re not going to treat them well…because they have the card. They can just go to another GP the next day [and] just pay 5 dollars.” (GP24)*

As compared to polyclinics, financial models encouraging transaction-based relationships were more likely to occur in GP settings, in particular participation in TPA and MCO administration disrupted the relationship between doctors and patients.

##### Drug formulary management and organisational prescribing know-how

Primary care clinics in Singapore both prescribe and dispense medications to patients at a single location and their operational models also determine the way drug formulary is managed in each clinic. The pharmacies within the polyclinics are managed by outpatient pharmacists and the drug formulary is controlled by the organisation. *Polyclinic doctors mentioned that they hardly made decisions on antibiotic procurement, which saved a lot of administrative load*. Patients were given scripts to collect antibiotic prescriptions from community or hospital pharmacies if the drug was not stocked up in the polyclinics:

*“There were some cases whereby a patient…[has] multiple allergies to different antibiotics and then the one that I wanted to give wasn’t available. Levofloxacin. So in that case I give him an external prescription that he can buy…in the other pharmacies.” (P6)*

On the other hand, *in GP settings, drug formulary management forms a large proportion of the GP doctors’ role in clinical practice, taking up time and effort, potentially distracting from time that could be given to patient care and relationship building.* As described by a couple of solo GP doctors:

*“We’re more than happy to lose the pharmacy actually…we have to manage the dispensary, to manage all these medications, [but] to us, this is not our core job right? Our core job is a doctor…to provide consultation and just charge the consultation…we can actually focus what is important to us.” (GP23)*

*“I have no problems with [abolishing dispensing role]. It reduces my headaches. I just put a consult fee and that’s it. I don’t need to buy drugs, and think about what tier I have, how many shall I stock, can I dispense it before the drug expires.” (GP24)*

On the other hand, *some GPs expressed that having control over customising their formulary and choosing antibiotic stock allowed them to improve knowledge of what was being dispensed and better ability to monitor and prescribe according to their clinical expertise and preferences*:

*“I own my practice…I can put very fanciful stuff…I mean we can order in. Private practice is very simple. You want something, it comes in 2 days. You don’t need a process of [procurement]. So it’s actually extremely minimal and efficient [when orders are placed].” (GP15)*

*“I have, over the years, kind of narrowed down my antibiotics to those that I most likely would use…I think I have almost never written a prescription outside for antibiotics.” (GP24)*

*“Before I start work anywhere, I will look at the stocks and see whether my favourite medicines are available or not. If they are not, I will ask for the medications to be brought in.” (GP27)*

Therefore, providing that drug formulary management was administrated such that it did not impinge on patient consultation times, there were benefits described by having control over this process. Though on the whole, outsourcing such processes were seen to help keep valuable patient consult time where it was most needed - with patients rather than procurement order sheets.

### Objective II: Exploring mechanisms that give rise to appropriate antibiotic prescribing across primary care settings

#### Mechanisms influencing practice

##### High patient loads, lack of continuity in care and the importance of trust building

*Trust between patients and doctors, and ability to leverage this to adequately communicate whether there was a real need for antibiotics was viewed as central to appropriate prescribing*. Relatedly, *high patient loads were said to impede this and the process of shared decision-making*. The lack of time, and likelihood of being able to see the same doctor and take time to counsel were traded-off to deal with the volume of patients:

*“Yeah, that…does play a factor. So let’s say if there [are] time constraints, sometimes I don’t have the luxury of time to explain in detail…So definitely…it will lead to more antibiotic prescription[s]…because we don’t have the time to explain in detail…so we end[ed] up giving more to those who insist[ed].” (P6)*

*“On days whereby I am superbly busy…sometimes you have to see like 80, 90 patients in a day, and if the patients request for antibiotics and you don’t have time…I guess if it’s so clear-cut that he needs it, I would just prescribe.” (GP13)*

In contrast, due to greater autonomy to control their patient flow, GPs in particular talked about ensuring that they spent sufficient consultation time to counsel each patient:

*“Another thing that we build in our practice [is that] we give time [to our patients]…[for] every patient, we schedule 10 minutes. So now we are quite happy that we have an appointment system.” (GP23)*

In certain settings, *discontinuity of attending doctors with regular patients, for example the registration system randomly assigning patients to an attending doctor, made trust building difficult*. In addition, regular rotation of doctors between the different care sections, such as acute walk-in, chronic care and paediatric sections impeded the doctors from delivering continuity of care to regular patients:

*“I mean usually [in the] polyclinic, there are a lot of patients. So our rapport is not as easy, I think. And we don’t usually see our own patients back. So maybe it’s not as easy for them to trust us.” (P4)*

This experience was different for many GPs. Due to the organisation’s valuing of autonomy and flexibility in shaping their practices in accordance to their personal values, solo and small group GP doctors expressed that they were able to structure their model of care to be conducive for the establishment of a trusting patient-doctor relationship. This happened, in part, through patient referrals as a basis for building a client base, and easy-to-use appointment systems ensuring continuity of care:

*“We have been around for a while and also our model of care is very different. So we go by appointment system. We do very little walk-in and a lot of people know us. They are referred by friends and all that. So after a while, the trust level is very high.” (GP14)*

*“We will keep out these doctor hoppers, because we have an appointment system... So those patients that are used to seeing us, they will book online and they have to pay five dollars to actually see us…once patients know that we practice in this way, and is very clinically based…they are very happy to come and…they are willing to pay.” (GP23)*

Overall, *it was emphasized that with continuity of care, which was enabled in more mature practices, came greater ease and opportunity to counsel patients on prudent antibiotics use*:

*“I do have a very matured practice, so I do understand where you’re coming from, whereby some patient[s] who say “Because you don’t give me antibiotics, you’re a lousy doctor, I’m not seeing you anymore, I go elsewhere”. So I actually do not have this problem…I would explain to the patients. And I do ask them, I don’t think you need antibiotics right now. You may later but you do not know. But if you can, it’s always better to avoid it, are you ok with it?...So usually [we are] able to come to a consensus.” (GP13)*

*“My patients are very well-selected…because over the years, you sort of train[ed] them not to use antibiotics…because we do have a reputation that we don’t give antibiotics, so generally after a while, all those in the neighbourhood [who] wants antibiotic…will not turn up in our clinic…it’s the training.” (GP14)*

In summary, practices that were able to emphasize trust building, manageable patient loads and continuity of care were able to catalyse optimal antibiotic prescribing practices.

##### Values and alignment of organisational culture with appropriate antibiotic prescribing

In addition, *it was notable that doctors who were able to align their personal values for patient-centred care over “service” provision with organisational culture, *and vice versa*, optimised appropriate antibiotic prescribing*. Such alignment was often complex and multi-factorial. For example, while solo and small group GP doctors bore more responsibilities to ensure business sustainability of their clinics, their clinics were also described as able to achieve greater individual autonomy and flexibility in aligning their practices in accordance to the personal values of their doctors. These GP doctors could often strike a balance between business concerns and freedom to practice their personal values on care delivery, and this included decisions on prudent use of antibiotics:

*“We charge very high for consultations…I do not need to sell antibiotics to earn money. I do not need to sell medicine to earn money…I can talk the whole half an hour with you and I charge 100 bucks. I don’t even need to…sell you anything. So that is the beauty of it. I am not pressured to give you antibiotics or for that matter, any medicine.” (GP14)*

*“In our practice we have a lot of control because basically we run our own practice…so basically we’re not obliged to follow what the patient wants. We are quite happy to lose the patient because we are so busy anyway. So we are not obliged to give whatever the patient requests for.” (GP23)*

Nonetheless, a couple of GP doctors shared how this context could enable other scenarios, depending on what was being prioritised and by whom. Sometimes the need to sustain business and the related value to optimise revenue could drive GP doctors towards prescribing antibiotics in order to increase earnings, e.g. prescribing either unnecessary or expensive non-generic brands to increase profit:

*“The principle behind every GP’s prescribing practice is different. I know that there are some more profit-driven GPs, whom I think would give antibiotics because of higher profit margins.” (GP28)*

*“I am sure there is financial pressure for doctors to add antibiotics…they may not disclose this. Because who will say that, “Oh I give medicine because it’s additional revenue but not because it’s indicated?” (GP29)*

Alignment of organisational and personal values that prioritise healthcare provision overriding business-centred models was clearly positioned as pathway to appropriate antibiotic prescribing outcomes.

##### Emphasis on liaison with patients and shared decision-making

That said, an important interim outcome was also identified. *Successful patient liaison was expressed as resulting from valuing and enabling shared decision-making:*

*“It is always important in family medicine that we establish a very close and long relationship with your patient. And a correct relationship is always a partnership. So when you have established a partnership, that means there is a great degree of trust and communication channels are naturally opened. So once that happens, it is very easy to be able to come up with a management plan that both agree on. And usually the patients would listen to the doctor.” (GP13)*

*“A good patient-doctor rapport will solve a lot of issues. One is trust, two is the willingness to work out problems together and solve it versus one who have no rapport where you are just there to ‘service’ the patients…[The patients] will treat you like a technician…but then again, rapport takes two hands to clap. So it is not just willing doctors but also the patient has to be willing to establish that. So overall, if there is cooperation, a lot of things tend to work out better, of course.” (GP30)*

The emphasis on cooperative patient liaison was positioned as driven by both organisational culture creating opportunity for this and a personal commitment for such exchanges which with the ultimate goal of allowing clinical judgment to prevail. Shared decision-making emerged as a clear interim outcome to enabling those related to appropriate prescribing.

##### Using data to monitor and evaluate appropriate antibiotic prescribing behaviours and related outcomes

*While audits were thought to be useful to improve antibiotic prescribing habits, primary care doctors expressed that more could be done*. In the polyclinics, regular audits were at times described as part of the organisational process for monitoring antibiotic prescribing, although these procedures were not often notable in practice:

*“So for us in the polyclinic…we do have check and balances on how we order our antibiotics.” (P19)*

*“I think polyclinics do audits on the antibiotics prescribing right? I think, I’m not that sure.” (P3)*

Similarly, in GP clinics, while random audits by the Ministry of Health were said to take place, antibiotic prescribing volumes were not queried during this process meaning that little external pressure to reduce antibiotic prescribing was being routinely applied and tracked:

*“We have audits…I mean MOH does come down and audit our medical records…They do look through medical records but they don’t go and count how many antibiotics you have used.” (GP30)*

*To enable successful antibiotic stewardship in the primary care setting, applied research needs to be conducted to provide a transparent platform for the formal use of monitoring and evaluation data to inform evidence-based guidelines*:

*“There should be an audit in every healthcare system whether it is a polyclinic, or a GP…Because we cannot be in every consult room every single time, so the only way to do is…to retrospectively audit the amount of cases where doctors are in a controlled environment like ours. In the polyclinic, it is very easy to do. I don’t know how feasible it is to do in the private practice but there should be some form of monitoring of this…you want to see whether in those situations, it was warranted for; and then the other thing is you can get patient’s feedback. I mean if there are more studies or surveys tracking patients in the private and polyclinic healthcare then we have a better idea of whether [antibiotic use] is really effective…or not in our population.” (P20)*

By having better transparency and agreed indicators that capture outcomes such as the core practice of shared decision-making known to influence appropriate prescribing, improvement on antibiotic prescribing in the primary care setting can be meaningfully driven forward.

### Objectives III: Building a conceptual model to inform planning and related strategies for targeting primary care service improvement for appropriate antibiotic prescribing

Taking a bird’s eye view, the operational models of primary care were tied to financing and ability to prioritise a more patient-centric approach reflected in deeply-rooted organisational structures and cultures that inform primary care provision. Primary care settings in the current study were demonstrably very heterogeneous environments, where doctors could be inspired to play either the role of service or healthcare providers. Thus, primary care doctors were observed to be driven in part by operational models. Those with stable government funding and centralized pharmacy for drug procurement and dispensing in publicly-funded polyclinics in Singapore, emphasized the importance of clinical consultations, keeping the focus on the patient as opposed to additional responsibilities.

**Fig. 1:**
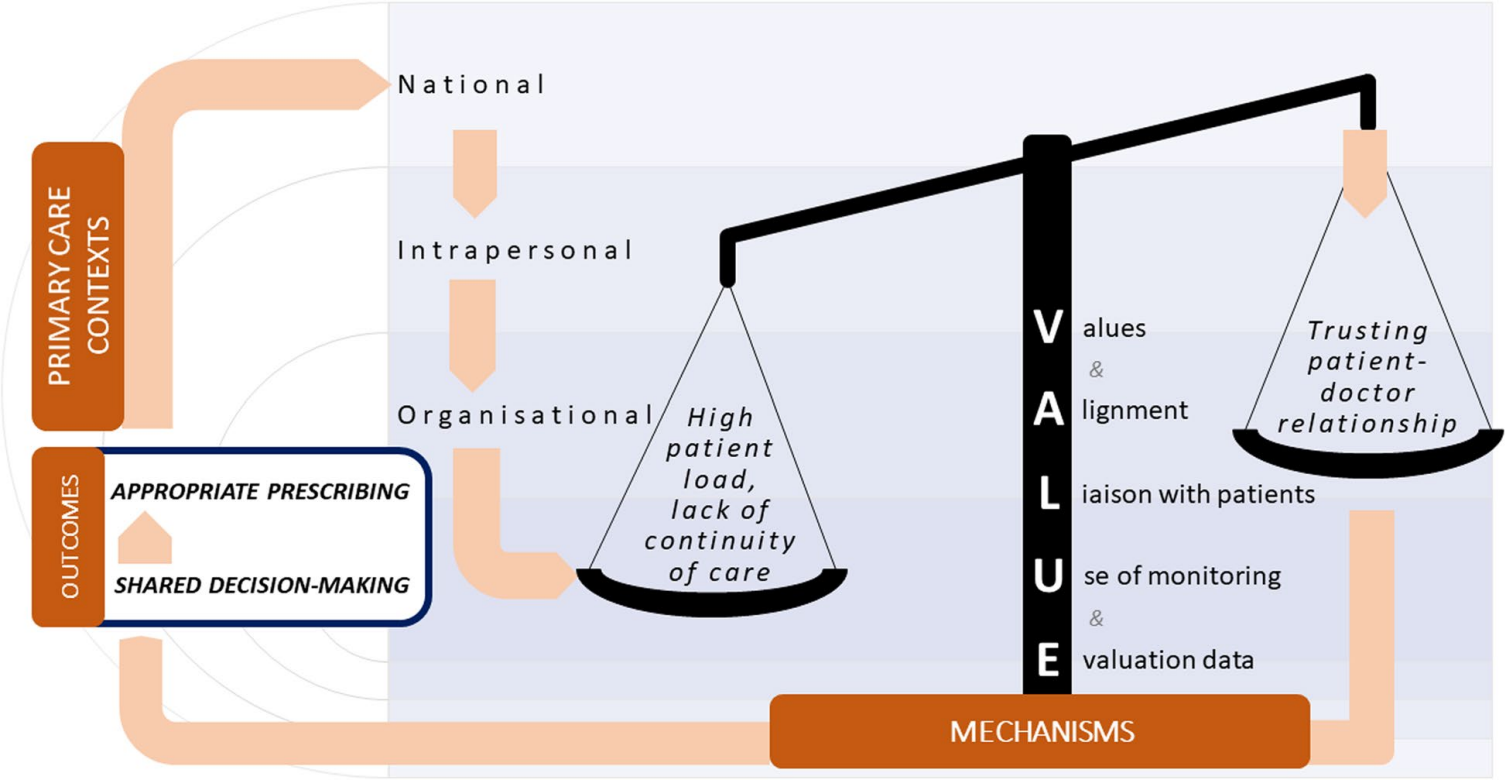
VALUE model for improving appropriate antibiotic prescribing

Models which can avoid having to bear with the consequences of not being able to build a mature practice/returning patient base, e.g. due to TPAs or MCOs, or be distracted from applying consistent antibiotic guidance and stewardship, or clear messages and consultation given to patients, will better allow inappropriate antibiotic prescribing to be avoided. In view of the above findings and known literature, we have proposed the VALUE model to conceptualise the key components of appropriate antibiotic prescribing and stewardship in the primary care (Fig. 1).

Across contexts, the following mechanisms were seen to leverage appropriate antibiotic prescribing, starting with encouraging holding ***V****alues* consistent with prioritising AMR reduction, and ***A****ligning* organisational culture to these, for instance by leveraging standardised guidelines and interpersonal intervention tools. Such interventions should account for the wider systemic constraints experienced in publicly funded, high patient turnover institutions, or private clinics with transactional models of care. Ultimately, a focus on ***L****iaison between patient and doctor* will drive better antibiotic prescribing outcomes.

For instance, building in adequate consultation time and props as discussion aids, or quick turnover communication tools in time-constrained settings. Message consistency will ultimately improve trust, helping to enable shared decision-making. Lastly, ***U****se of monitoring data to track* and ***E****valuate* antibiotic prescribing using meaningful indicators, that account for the role of shared decision-making can also be leveraged for change*.* Exemplar intervention types and related change strategies aligning to the VALUE model are presented in Table 2. The VALUE model is recommended to guide intervention planning and it is not intended to be static reflection of optimal antibiotic prescribing, but as a summary of findings, reflecting a description of what is currently known [43]. It is proposed as an implementation too, components of which can be tested empirically and updated accordingly.

**Table 2:** Exemplar organisational and behavioural change strategies corresponding to the VALUE model for improving appropriate antibiotic prescribing

**Mechanisms**	**Organisational and behavioural change strategies**
**V**alues and **A**ligning	*Message consistency:* • Standardised guidelines and agreed protocols in use across primary care sectors *Alignment between organisation and doctors:* • Organisational-led email circulars leveraging these, sent in the name of established and valued institutional bodies • Reminders of the risks and impact of AMR in primary care practice, while also promote the need to make time or build in micro-strategies, especially in clinics with high patient load, for discussion on appropriate antibiotic prescribing, and the risks of inappropriate prescribing • Promoting shared decision-making through incorporation into clinics’ mission statements and inculcating such values to newly employed doctors during orientation, and utilise trigger videos which use patients’ experiences as discussion points, especially in larger clinics • Informal training to promote role modelling of appropriate patient counselling using mentorship programming for junior doctors *Alignment between doctors and patients:* • Organisational promotion of continuity of care, encouraging patient ‘loyalty’, and delivering care through a fixed team of doctors such that understanding of antibiotic prescribing decisions can be shared and sustained over time
**L**iaison with patients	*Communication aids:* • To improve dealing with high patient loads through fast turnover communication tools (micro-strategies) such as decision aids with consistent messaging • Gamification strategies to empower public to make shared decision-making through gaming interventions aimed at engaging both patients (playing the game) and doctors in discussion about the takeaway messages from the game together
**U**se of monitoring data to track and **E**valuate	*Audit and feedback:* • Use of routine monitoring data for audit and feedback on prescribing behaviours at the individual doctor level (closed feedback) and to benchmark organisations against one another (open feedback) shared at regular meetings or in email circulars • Distil research findings on AMR and inappropriate antibiotic prescribing into short, accessible briefs posted and disseminated in clinical settings (knowledge management practices) *Developing meaningful indicators* • Incorporate shared decision-making as a key indicator measuring appropriate antibiotic prescribing • Measure and evolve elements of the VALUE model based on empirical evidence

## Discussion

Our study has identified themes that lend critical insights on antibiotic prescribing by primary care doctors and shed light on the underlying mechanisms driving antibiotic prescribing across primary care settings. Themes relating to the operational models used in clinics, financial considerations, drug formulary management, patient load, and a trusting patient-doctor relationship, were demonstrated as central to appropriate antibiotic prescribing.

The importance of such elements have been highlighted elsewhere [7]. For example, both the importance of established and up-to-date national antibiotics guidelines [31] and that of valuing patient-centred care have been demonstrated to contribute to reducing antibiotic prescribing [8]. In addition, the role of the interpersonal level is known to affect antibiotic prescribing, in particular when doctors do not take time to accurately assess patients’ expectations of antibiotic prescribing [44]. Further to which, it is notable that being given antibiotics does not always correlate with satisfaction of the clinical encounter anyway [44, 45]. As for use of monitoring and evaluation data, this has long been recommended for practice improvement by the US CDC guidelines and recently, by Arieti *et al*. [5, 46].

Nevertheless, there remains a powerful need to ‘connect the dots’ by providing a realist [42], applied and evidence-based conceptual model that maps the social ecology and potential areas for intervention to improve appropriate antibiotic prescribing. A recent study conducted in Sweden found that doctors in private practice were 6% more likely to prescribe antibiotics as compared to doctors in public practice [47], with a similar trend observed from another cross-sectional study conducted in Malaysia [48]. Instead of dissecting and addressing the issue of inappropriate antibiotic prescribing by different primary care funding structures, the current study offers a comprehensive exploration across private and public sectors. Outlining not simply the elements driving appropriate and inappropriate practice but how these interrelate.

Collective and coordinated antibiotic stewardship efforts in primary care (both public and private practice) would improve appropriate antibiotic prescribing in primary care clinics at a national level [49]. Our study highlighted opportunities for national interventions to improve antibiotic prescribing in primary care, particularly in private practices which manages the bulk of primary care acute conditions in Singapore. It has been observed that primary care doctors desired national guidelines on antibiotic prescribing to standardise best practices [31]. Guidelines based on local epidemiological data and antibiotic susceptibility patterns would be crucial for supporting primary care antibiotic stewardship and overcoming variations in context- and value-based prescribing practices. Clinical decision support tools can also play a role in guiding primary care doctors in evidence-based antibiotic prescribing decisions by developing risk prediction models to guide antibiotic prescribing decisions, as demonstrated in a local outpatient emergency department setting [50].

The primary strength of the current study lies in the construction of the VALUE model and its transferability to other primary care contexts. The conceptual model can be cohesively applied to evaluate each level of the ecosystem to address inappropriate antibiotic prescribing. For instance, the VALUE model can be used to assess organisational culture, personal motivations, to critique and overhaul operations while accounting for funding structures, and helping to refocus where and how time is spent when battling high patient loads. The study also undertook one-on-one semi-structured interviews with doctors in primary care settings which allowed an in-depth exploration of antibiotic prescribing practices among this unique group of healthcare workers. Moreover, the researchers were careful in building rapport with study participants, which enabled them to be forthcoming and open in sharing their practices and experiences. Additionally, the use of a maximum variation purposive sampling strategy enabled the study to elicit the broadest range of experiences within our sample of interest. The study also utilised principles of data saturation and intercoder agreement to ensure the rigour and trustworthiness of the study findings.

We acknowledge that our data is however limited to a context and primary care practice that were pre-COVID-19. Rapid shifts may be on their way to influence the ecology of acute respiratory tract infections in the last 12 months.

## Conclusion

Multiple factors influence antibiotic prescribing in primary care. The ability to make shared decisions with patients on antibiotic prescribing is dependent on the balance between managing patient load, continuity of care or ‘loyalty’ of returning patients, and building trusting patient-doctor relationships. Systemic constraints and factors hindering interpersonal interactions with patients can be overcome by aligning values on reducing AMR and promoting patient liaison through consistent messaging and tailored intervention tools improving patient liaison. Antibiotic stewardship interventions will be rendered more effective if monitoring and evaluation data are used to capture indicators that are known to effect change, and these shared meaningfully and transparently to both inform audit and feedback strategies and to updated theory and evidence base of intervention initiatives.

## Supplementary Information

**Additional file 1.** Topic Guide: Understanding antibiotic prescribing through a multilevel approach.

## Data Availability

The datasets used and/or analysed during the current study are available from the corresponding author on reasonable request.

## Abbreviations

AMR: Antimicrobial resistance
CDC: Centers for Disease Control and Prevention
GP: General practitioner
MCO: Managed care organisations
TPA: Third-party administrator
