# Supplementary material for: Exploring antibiotic prescribing in public and private primary care settings in Singapore: a qualitative analysis informing theory and evidence-based planning for value-driven intervention design
Source: BMC Fam Pract. 2021 Oct 15;22:205. doi: 10.1186/s12875-021-01556-z (PMC8519324; doi:10.1186/s12875-021-01556-z)
Supplement: Supplementary file 1 — Additional file 1. Topic Guide: Understanding antibiotic prescribing through a multilevel approach. [file 12875_2021_1556_MOESM1_ESM.docx]

**Understanding antibiotic prescribing through a multilevel approach**

Objectives:

1. Ascertain perception towards antimicrobial resistance and appropriate prescribing among physicians in primary care clinics;
2. Identify clinical and organizational determinants for antibiotic prescribing among physicians and peer relations;
3. Discover the relationship between patients and healthcare providers around antibiotic prescribing;
4. Identify ways to improve awareness and optimal use of antibiotics

Topics:

1. Intrapersonal: Perception towards antibiotic resistance and appropriate prescribing in primary care settings
2. Organizational: Role of organization and peer relations in determining antibiotics prescribing
3. Interpersonal: Relationship and Interaction with patients when prescribing antibiotics
4. National: Ways to improve awareness of antibiotic use and antibiotic resistance
5. **Perception towards antibiotic resistance and appropriate prescribing in primary care settings**

- How would you describe antibiotic resistance?
  - Would you mind describing how you think antibiotic resistance is an issue to the healthcare system?
  - How do you think the primary care sector is involved in the antibiotic resistance issue and why?
  - Would you describe some situations which you feel empowered or not empowered in playing a role in reducing antibiotic resistance in Singapore?
- Please describe what are the patient and personal factors which determines your decision to prescribe or not prescribe antibiotics to a patient.
  - How and where did you gather such prescribing expertise?
- Would you mind describing what are some facilitators and barriers affecting the successful management of antibiotic resistance in the primary care sector?

1. **Organizational determinants for antibiotics prescribing**

- What are the various types of antibiotics available in your clinic?
  - Could you share with me the 3 most common antibiotics you usually prescribe?
    - How do you decide which type of antibiotics at which dose to prescribe to which patients or conditions?
    - Would you describe some situations when your clinical expertise does not match with the formularies available?
- What do you know about delayed antibiotic prescribing or standby antibiotic prescribing?
- Would you please describe your daily patient load and roughly what proportion of your patients, with an infection condition, required antibiotics?
  - Would you mind describing any situations which your patient load had affected your antibiotic prescribing practices?
    - Could you also share how seeing adults and children (as accompanied with parents/guardians) affect your antibiotic prescribing practices differently?
- Would you describe situations when a decision to prescribe antibiotics was hard to make and what did you do in these situations?
- How would you consult your peers or colleagues?

1. **Relationship and interaction with patients when prescribing antibiotics**

- In general, how do you think that physician-patient relationships can affect antibiotic prescribing?
  - Could you share further how seeing regular patients and first-timers affect your antibiotic prescribing practices differently?
  - Would you mind describing some difficult situations you have faced with your patients which are related to antibiotic prescribing and how did you feel during those situations?
    - What happened in the end and how did the incident(s) affect you?
- Could you describe situations when you discuss with your patients on antibiotics?
  - How knowledgeable do you think that your patients are on the use of antibiotics and why do you say so?
    - Are there patient profiles where you recognise an inclination to err on prescribing or on not prescribing antibiotics?
  - How do you think their knowledge of antibiotics shapes their expectations for antibiotics when they enter the clinic?
    - Would you share some instances when your patients negotiate with you on antibiotics?
      - How do you manage when they strictly demand for antibiotics?

1. **Ways to improve awareness of antibiotic use and antibiotic resistance**

- How would you describe the current knowledge, perceptions and attitudes primary care physicians and the members of the public have in terms of antibiotics use and antibiotics resistance?
  - What are some gaps that can be explored to further improve antibiotic use?
- In November 2017, Singapore’s Ministry of Health announced a National Strategic Plan on Antibiotic Resistance. Are you aware of it?
  - If yes, would you like to share your thoughts on the national plan?
  - If no, explain: It is a national movement by the government to acknowledge a rising global health issue and Singapore has pledged to do her part in this battle against antibiotic resistance. The National Strategic Plan hopes to reduce the emergence and prevent the spread of drug-resistant microorganisms through the five core strategies such as research, education, optimising antibiotic use, surveillance, and infection prevention and control.
    - After hearing this, what are your thoughts on the national plan?
- We have come to the end of the interview. Thank you for participating in this session and I really appreciate your time spent here today. Do you have anything more to add to this discussion or have any questions to ask?

- End -
